# Chitosan/Virgin-Coconut-Oil-Based System Enriched with Cubosomes: A 3D Drug-Delivery Approach

**DOI:** 10.3390/md21070394

**Published:** 2023-07-06

**Authors:** Simone S. Silva, Luísa C. Rodrigues, Emanuel M. Fernandes, Diana Soares da Costa, Denise G. Villalva, Watson Loh, Rui L. Reis

**Affiliations:** 1 3B’s Research Group—Biomaterials, Biodegradables and Biomimetics, University of Minho, Headquarters of the European Institute of Excellence on Tissue Engineering and Regenerative Medicine, AvePark, Zona Industrial da Gandra, 4805-017 Guimarães, Portugal; luisa.rodrigues@i3bs.uminho.pt (L.C.R.); efernandes@i3bs.uminho.pt (E.M.F.); diana.costa@i3bs.uminho.pt (D.S.d.C.); rgreis@i3bs.uminho.pt (R.L.R.); 2 ICVS/3B’s—PT Government Associate Laboratory, 4806-909 Braga/Guimarães, Portugal; 3 Institute of Chemistry, University of Campinas (UNICAMP), Campinas 13083-970, Brazil; denise.villalva@gmail.com (D.G.V.); wloh@unicam.br (W.L.)

**Keywords:** chitosan, virgin coconut oil, cubosomes, emulsion system, porous structures, biomedical applications

## Abstract

Emulsion-based systems that combine natural polymers with vegetable oils have been identified as a promising research avenue for developing structures with potential for biomedical applications. Herein, chitosan (CHT), a natural polymer, and virgin coconut oil (VCO), a resource obtained from coconut kernels, were combined to create an emulsion system. Phytantriol-based cubosomes encapsulating sodium diclofenac, an anti-inflammatory drug, were further dispersed into CHT/VCO- based emulsion. Then, the emulsions were frozen and freeze-dried to produce scaffolds. The scaffolds had a porous structure ranging from 20.4 to 73.4 µm, a high swelling ability (up to 900%) in PBS, and adequate stiffness, notably in the presence of cubosomes. Moreover, a well-sustained release of the entrapped diclofenac in the cubosomes into the CHT/VCO-based system, with an accumulated release of 45 ± 2%, was confirmed in PBS, compared to free diclofenac dispersed (80 ± 4%) into CHT/VCO-based structures. Overall, the present approach opens up new avenues for designing porous biomaterials for drug delivery through a sustainable pathway.

## 1. Introduction

Natural polymers and bioactive compounds of marine origin have emerged as sustainable sources that are able to develop high-value systems of biomedical interest as pharmaceutics, nutraceuticals, cosmetics, and biomaterials [1,2]. In this context, the use of marine-origin polymers—alginate, chitin, chitosan, and fucoidan, obtained from marine biomass—in the development of scaffolds has drawn significant attention as an eco-friendly and sustainable strategy [1,3]. Natural polymers offer inherent biocompatibility, biodegradability, and lower toxicity, with the potential for cell-specific interactions, while synthetic polymers provide better control over characteristics, stability, and functionalization [4]. Then, the choice between them depends on the specific requirements of the drug-delivery system, including the drug’s characteristics, target site, intended release profile, and potential immunogenicity concerns. Moreover, porous structures that are produced using natural or synthetic polymers offer significant advantages as cell-supporting systems for seeding cells in vitro and for use as drug-delivery devices—including high-drug loading capacity, controlled and targeted drug release, biocompatibility, protection, and drug stabilization [5,6]. These advantages could lead to more effective and friendly drug-delivery systems, enhancing therapeutic outcomes and patient quality of life. 

Chitosan (CHT), a natural polymer derived from the chitin found in crab shells, has been the subject of intensive research, due to its vast number of physicochemical and biological activities [3,7]. CHT has a structure composed of repeating units of N-acetyl-2-amino-2-deoxy-d-glucopyranose (acetylated unit) and 2-amino-2-deoxy-glucopyranose (deacetylated unit), which are linked by β-(1→4)-glycosidic bonds [3]. It displays interesting physical, structural, and chemical features, which result in film-forming ability, antibacterial action, biocompatibility, and biodegradability [3,7,8]. Moreover, CHT’s cationic nature encourages the interaction of the available functional groups in its structure with different molecules, polysaccharides, and proteins, opening possibilities for designing transdermal drug-release systems, wound dressings, in vitro models, and other tissue-engineering applications [3,9,10,11,12]. 

Further research has shown that combining CHT with vegetable oils, such as copaiba oil [13], olive oil [14], and virgin coconut oil (VCO) [11,15], produces emulsion-based systems as sustainable and environmentally friendly alternatives to synthetic oils. Such systems have the advantage of combining the inherent beneficial properties of vegetable oils, such as antioxidant and anti-inflammatory effects, with the intrinsic features of CHT to produce systems with increased and controlled release properties, among other features. The positive health effects of these systems also increase their potential uses, ranging from food to biomedical uses [11,13,14,16]. 

In our recent work [11], CHT and VCO, a renewable extract from coconut kernels [17] were combined to obtain emulsion films. In these films, positively charged amino groups of CHT and the negatively charged free fatty acids from VCO interacted within the emulsion system through coulombic forces. The CHT/VCO-based emulsion films were characterized as superabsorbent materials, and they were not harmful to human adipose stem cells (hASCs), allowing good cell viability without associated cellular damage. This knowledge about CHT/VCO-based emulsion systems enabled us to explore them as drug-delivery vehicles.

On the other hand, cubosomes are types of liquid crystalline nanoparticles developed mostly from amphiphilic lipids, particularly glyceryl monooleate or phytantriol (a cosmetic component), with the addition of a suitable stabilizer [18]. Due to their substantial hydrophobic region, cubosomes have a liquid-crystalline structure and offer a significant advantage over other particles, such as liposomes. This unique characteristic enables them to have a higher loading capacity for hydrophobic drugs, while allowing the incorporation of hydrophilic drugs [19]. Previous studies used phytantriol cubosomes with incorporated diclofenac, which served as a model drug [20]. 

Beyond chemical compounds, cubosomes also present the potential for delivering various proteins into biological systems [18]. In recent approaches, cubosomes have been presented as promising lipid-based nanosystems for sustained drug delivery and for the enhanced oral bioavailability of drugs with potential applications in skin diseases and transdermal, ocular, and chemotherapy drug delivery [20,21,22,23,24]. In addition, introducing cubosomes into CHT solutions may further enhance their adjuvant effect [25]. 

In this work, we explored a CHT/VCO-based emulsion system to create 3D architectures using a freeze-drying technique and the incorporation of cubosomes loaded with sodium diclofenac to develop a drug-delivery system. For this purpose, phytantriol cubosomes (Cub) were prepared with sodium diclofenac (SD), a non-steroidal anti-inflammatory drug that is frequently used to treat inflammatory diseases [26,27], and were chosen as a model drug. With this proposed approach, we hypothesized that the CHT/VCO-based system would provide controlled release properties, while protecting the cubosomes from degradation. Evaluations of the produced structures’ attributes, mechanical stability, and swelling behavior, together with an evaluation of the release profile of the system, were performed to evaluate the potential of this system.

## 2. Results and Discussion

New avenues and innovative therapeutic approaches for treating diseases have been made possible by rapidly expanding the biomedical use of polymers and proteins produced from natural sources. In fact, the inherent activities of marine-derived polymers, such as CHT—due to its sustainability, biocompatibility, and biodegradability—have allowed the development of promising drug-delivery systems. The CHT-based systems—gels, micro/nanoparticles, films, and scaffolds—have been successfully developed for transdermal, nasal, oral, and ophthalmic administration, providing a sustained release of drugs and bioactive agents [5]. In addition, CHT can enable the interaction between the drug carrier and the biological target, exhibiting biocompatible features [28]. 

Recent research has also shown that combining CHT with vegetable oils such as VCO is a viable way to create matrices with improved performance [11,15], as VCO has proven health benefits, including anti-inflammatory, antioxidant, and analgesic properties [29,30]. In addition, those previous studies indicated that the compatibility of VCO with CHT and the modulation of their properties could be achieved using low concentrations of VCO [11,15]. The CHT/VCO-based emulsion demonstrated high stability that would allow the inclusion of small molecules following its production under mild conditions. 

In this work, CHT/VCO-based 3D architectures were developed using sustainable procedures involving the emulsion system (CHT and VCO) and the incorporation of cubosomes doped with sodium diclofenac, followed by freeze-drying. These CHT/VCO-based structures are referred to as CV-based structures. To build up the CV-based system, it was necessary to study the ratio used by each component to produce the developed scaffolds, which was optimized in preliminary tests. Finally, the combination of CHT (3% *v*/*w*), VCO (1% *v*/*v*), glycerol (1% *v*/*v*), and Cub (1% *v*/*v*) was chosen as a representative structure for drug delivery.

Before being incorporated into the CV system, the cubosomes (Cub) were characterized. The developed Cub presented a monomodal particle-size distribution with mean diameters ranging from 187 nm (CV_CubD) to 196 nm (CV), based on intensity measurements via SAXS analysis. These results suggested that adding 10 wt.% diclofenac promotes a slight decrease in the mean diameters observed for the cubosome dispersions, which was similar to the results of previous reports [20]. The efficiency of diclofenac encapsulation (EE %) in cubosome dispersions was 96.5 ± 0.1 wt.%, confirming its effective incorporation. This result was close to the results reported in other studies, where the incorporation of diclofenac in bilosomes, as a vesicular carrier, presented EE% with maximum values of 93.2 ± 2.21% [31].

### 2.1. Structural and Morphological Features

**Figure 1:**
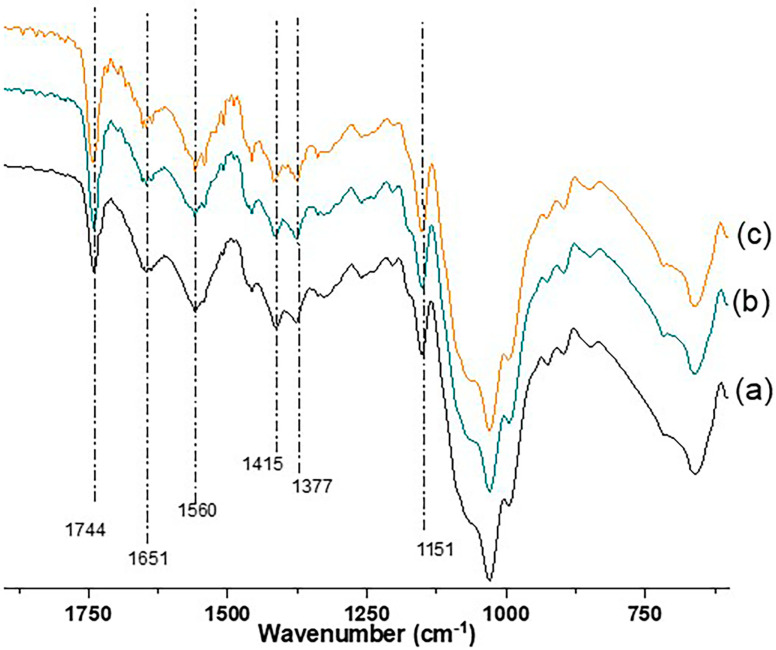
FTIR spectra of the developed CV-based scaffolds: (**a**) CV, (**b**) CV_Cub, and (**c**) CV_CubD.

The structural differences between CV-based emulsion structures with and without cubosomes were analyzed by FTIR, as shown in Figure 1. The achieved spectra showed prominent peaks that were consistent with the VCO presence at 1744, 1459, 1377, and 1156 cm^−1^, corresponding to the stretching of the carboxylic group (C = O), the bending of methylene (CH_2_), the bending of methyl (CH_3_), and the stretching of esters (C-O), respectively [15,32]. In addition, the presence of CHT was confirmed, with peaks at 1650 cm^−1^, corresponding to the acetylated amino group of CHT (−NCH = O) stretching of amide I, and around 1560 cm^−1^, corresponding to the stretching of amide II (NH_2_) [33]. Moreover, no significative differences were observed between the doped (CV_CubD) and undoped (CV_Cub) architectures. This suggests that no significant interactions were established between the emulsion solution and those with dispersed cubosomes with and without the drug. We also suggest that the reduced amount of cubosomes added to the CV-based system makes it difficult to detect their characteristic peaks, which may be occulted due to the much higher scattering intensity of the CHT and VCO characteristic ones.

In addition, no significant shifts were observed for the reference peaks of the CV-based system, suggesting that the chemical changes that were introduced did not affect the stability of the previous interactions or the chemical environment of the supporting emulsion components. These interactions maintaining the coulombic forces between the positive amino groups of CHT and the negatively charged free fatty acids from VCO, as well as the ion-dipole and H-bonds between the carboxyl group of the fatty acids and the protonated (NH_3_^+^) and non-protonated (NH_2_) amine group of CHT, as reported earlier for a similar emulsion-based system [11].

**Figure 2:**
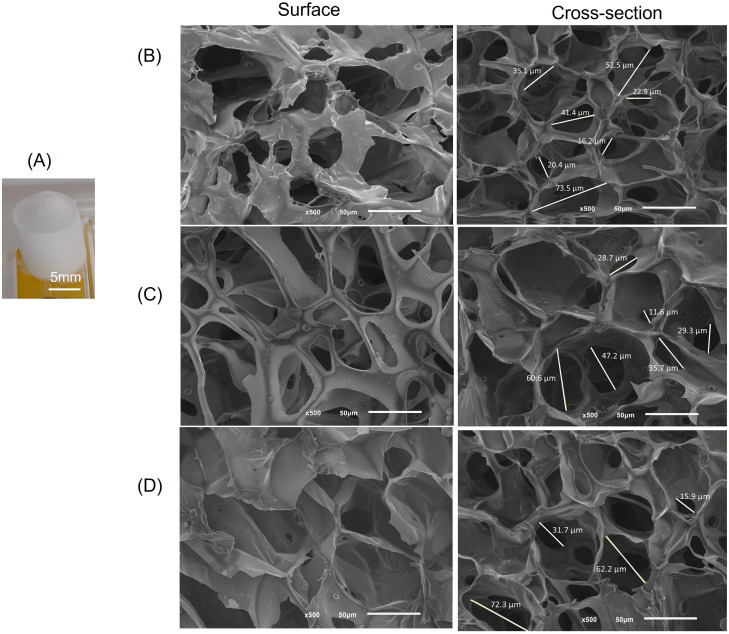
Appearance of the produced cylindrical scaffolds (**A**) and SEM microscopy images of the surface and cross-sections of the CV-based scaffolds; (**B**) CV; (**C**) CV_Cub; and (**D**) CV_CubD. Scale bar 50 µm and a magnification of 500×.

SEM was used to examine the surface and cross-section morphology of the CV-based scaffolds (Figure 2). According to the SEM micrographs, all the scaffolds developed a porous structure, which would meet the cells’ needs for nutrients and material exchange. Generally, both CV and CV_Cub seemed to present a high number of open pores compared to CV_CubD. The internal pore size in the cross-sections showed values ranging from 20.4 to 73.5 µm (CV), 22.7 to 61.7 µm (CV_Cub), and 19.4 to 72.2 µm (CV_CubD). 

Typically, CHT scaffolds produced by freeze-drying have well-defined lamellar-type morphologies [33,34], and they must be neutralized with sodium hydroxide solution to stabilize their structures. In earlier research [11], it was shown that specific interactions between the hydrophobic component (VCO) and the hydrophilic component (CHT) promoted the stabilization of their structure. This behavior was also confirmed on CV-based scaffolds, where the neutralization step was unnecessary after structure formation, even when CHT was selected in high concentration (3% *v*/*w*), which was optimized in preliminary swelling tests. According to the literature [35], the dispersed aqueous phase in emulsion-based systems is subjected to high shear stresses to disperse water as droplets in the oil phase, and vice versa, reducing the droplet size. Therefore, we hypothesized that the differences in morphology (internal pore size) in the CV-based scaffolds could be related to CHT concentration and its interactions with various system components in the emulsion system.

### 2.2. Mechanical Properties

**Figure 3:**
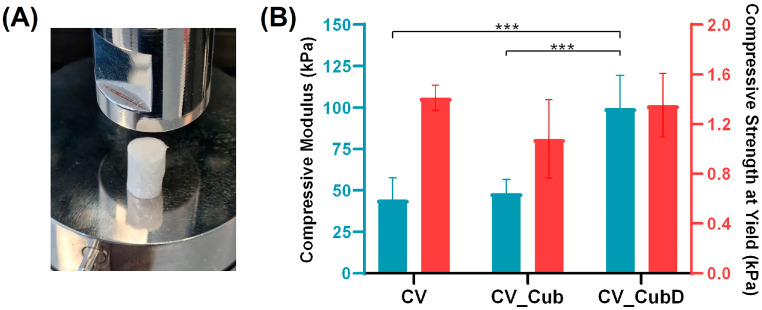
Mechanical properties of the scaffolds under compression load with 6 mm of diameter: (**A**) representative scaffold incorporating doped cubosomes before uniaxial compression test, and (**B**) compressive modulus and compressive strength at a yield at offset strain of 0.2%. The significance levels between groups was *** *p* < 0.001.

The mechanical behavior of the developed scaffolds was evaluated under a uniaxial compression load, and the results are reported in Figure 3. The obtained stiffness of the developed scaffolds was in the range of 44.5 ± 13.1 kPa for the CV, 48.3 ± 7.4 kPa for the CV_Cub, and 99.6 ± 19.8 kPa for the CV_CubD. The significant increase in the compressive modulus in the presence of the drug might occur because diclofenac has a lipophilic nature, so it prefers the oil phase (VCO), which may result in a structural rearrangement between the polymeric and the oil phase. In addition, the morphology of the cross-section results for the CV_CubD showed a high number of small pore sizes (Figure 2D), and this scaffold architecture contributed to the increase in the stiffness. 

Some previous studies provided important insights into understanding subtract stiffness on cell response [36,37]. They found that increasing the surface stiffness of biodegradable polymeric systems could positively influence their cell metabolic activity [36]. The yield strength corresponds to the yield point at which there is a transition from elastic to plastic behavior. The obtained values are almost in the same range, with a mean value of 1.08 kPa for the CV_Cub, followed by 1.35 kPa for the CV_CubD and 1.41 kPa for the CV scaffold, respectively. Regarding the yield strength, no significant statistical differences were found between conditions. This result demonstrated the potential of loading the porous architectures with diclofenac, without compromising the mechanical stability of the scaffolds.

### 2.3. Stability and Degree of Swelling

**Figure 4:**
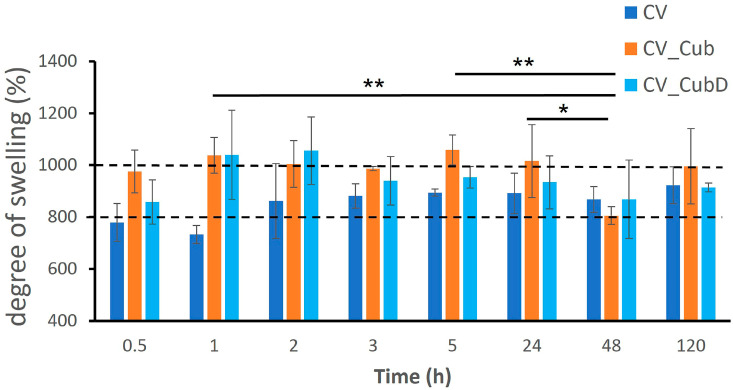
Degree of swelling of the CV-based scaffolds immersed in PBS. The significance levels between groups were * *p* < 0.05 and ** *p* < 0.01. The dashed lines meaning the two-maximum degree of swelling for the different formulations for comparison proposes.

To understand the stability of the CV-based scaffolds at different pH solutions, pH 5 and pH 7.4 (PBS), the swelling behavior was assessed (Figure 4 and Figure S1). It was observed that after a short period immersed in PBS (30 min), all formulations absorbed several times their weight reaching maximum values of 780% (CV), 975% (CV_Cub), and 860% (CV_CubD) for PBS (Figure 4), and 865% (CV), 890% (CV_Cub), and 800% (CV_CubD) for pH 5 (Figure S1). This trend was confirmed after a prolonged immersed time (up to 120 h) for both pHs. Statistical differences were found for the degree of swelling of CVC after 1 h and 48 h in PBS, while no statistical differences were found between the formulations after immersion in pH 5 (Figure S1). 

CHT-based scaffolds swell at low pH (acidic medium), due to the protonation of their amino/imine groups, where the protonated positively charged moieties on the polymer chains repel one another, which results in swelling [38]. Neutralization or crosslinking is typically utilized on CHT scaffolds to reduce their swelling. Alternatively, the presence of glycerol in CHT solutions can lead to intra- and intermolecular glucosamine interactions [39,40], modulating their swelling. 

In our earlier studies [11], the association of CHT and VCO in the emulsion system led to the superabsorbent behavior of CHT/VCO-based films, whereas the addition of glycerol controlled the swelling. To reduce swelling, glycerol (1% *v*/*w*) was added to the CV system. Overall, the protonation of amino groups (CHT), glycerol, and the morphological characteristics of the 3D matrices could work together to explain the behaviour of the CV-based scaffolds.

### 2.4. Release Study

**Figure 5:**
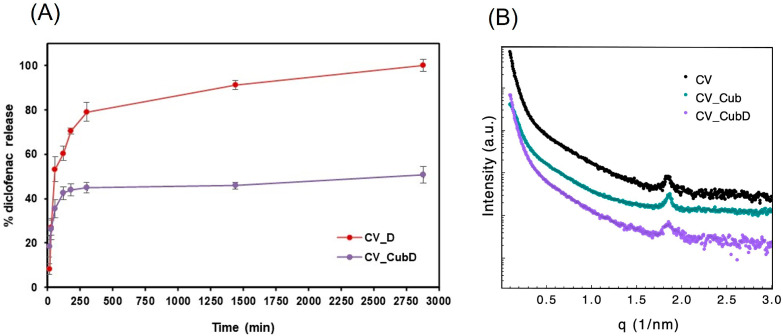
CV-based scaffolds analysis: (**A**) Drug-release behavior of the CV_D and CV_CubD scaffolds immersed in PBS and (**B**) SAXS curves of the CV (black squares), CV_Cub (green circles), and CV_CubD (purple circles) scaffolds.

The cubosomes dispersions previously incorporated in the scaffolds were released from the scaffolds after immersion in PBS (Figure 5). The aliquots collected after 48 h of releasing were analyzed by DLS, and the mean diameter results were 550 ± 37 nm and 748 ± 90 nm for empty and diclofenac-loaded formulations, respectively (Table 1). The obtained results show that the collected nanoparticles were larger than the original cubosomes in dispersion, suggesting general changes in the nanoparticles due to the interaction with the emulsion matrix.

According to the data presented in Figure 5A, the loaded diclofenac, used as a model drug, was successfully released from the architectures when dispersed as a free drug or encapsulated into cubosomes. Like the earlier observed profiles envisioning diclofenac release from hyaluronic acid hydrogels [20], the free diclofenac was delivered faster than diclofenac encapsulated into cubosomes on emulsion-based architectures. After 5 h of immersion in PBS, a plateau was reached, with an accumulated release of 45 ± 2% for encapsulated diclofenac in the cubosomes and 80 ± 4% for free diclofenac dispersed in emulsion-based structures. This trend suggests that the emulsion’s lipophilic nature favors the drug’s interaction and its release to the media in free and encapsulated diclofenac-loaded structures. Furthermore, the suggested interactions could protect the encapsulated drug, avoiding its degradation in a physiological environment. Therefore, many lipophilic and anti-inflammatory drugs that could be utilized to treat chronic diseases will benefit from those proposed systems’ features.

The release efficiency from the immobilized cubosomes in emulsion-based structures is higher than in the earlier-reported hyaluronic-acid-based hydrogels [20]. This observation, combined with the DLS (Table 1) and SAXS (Figure 5B) results, suggests that the liquid-crystalline structure of the loaded cubosomes dispersed into the emulsion-based architectures may be disrupted. This is also supported by the absence of the characteristic Bragg peaks on the SAXS spectra, which may justify a more significant release of the cubosomes-encapsulated drug to the media, over time, in the emulsion-based proposed structures.

The expected Pn3m symmetry of the cubosomes and their characteristic peaks’ distances [41] were not identified when incorporated into the scaffolds (Figure 5B), and all the curves showed peaks at 1.84 nm^−1^ that correspond to 3.4 nm of distance commonly related to micellar structures of approximately 12 nm in diameter, which were not detected by DLS. The absence of the peaks associated with cubosomes in SAXS curves suggests that the amount of 1% (*v*/*v*) of cubosomes incorporated in the scaffolds was not enough to produce detectable SAXS peaks.

Another possible explanation for the absence of the characteristic Bragg peaks, based on the literature [42], is the migration of fatty acid molecules (the majority of lauryl acid) from the CV emulsion to the cubosomes, causing changes in their liquid-crystalline structures [43]. The combined results of SAXS and DLS (Table 1) confirmed the nonexistence of phytantriol cubosomes with Pn3m symmetry [42,43]. The eventual changes were promoted by material transfer in the cubosome-emulsion mixtures, as mentioned in the literature [43], which could control the oil micelles’ sizes by cubosomes, depending on the n-alkane chain length of the fatty acid. In the case of short chains, such as lauryl acid, their migration from oil micelles into cubosomes is expected, increasing the nanoparticle sizes and decreasing the oil micelles. 

**Figure 6:**
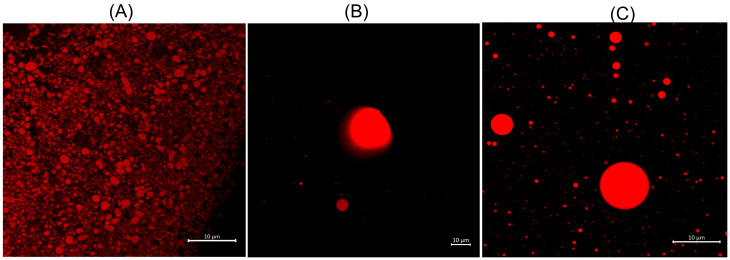
Confocal images of the systems: cubosomes (**A**), CV emulsion (**B**), and CV_Cub-based emulsion (**C**). All systems were stained with nile red. Scale bar: 10µm.

To address this hypothesis, the cubosomes and CV-based systems (CV and CV_Cub) were stained with Nile red (NR) [44], which was able to interact with the VCO droplets and cubosomes, emitting fluorescence and allowing us to examine the size of the cubosomes and oil droplets. The confocal analysis of the systems (Cub, CV, and CV_Cub) demonstrated that oil droplets decreased in size when cubosomes were dispersed in the solution (Figure 6), which was especially noticed for the larger droplets: for cubosome-loaded emulsions, the maximum diameter observed was 11.5 µm, while in emulsion without cubosomes, the bigger droplets presented diameters of around 25 µm. Nevertheless, different droplet sizes were observed in both emulsion samples, ranging from 0.3–25 µm for the emulsions without cubosomes and 0.9–11.5 µm for the emulsions with cubosomes. Considering the NR-loaded cubosomes that are present in the emulsion, an inverse behavior was observed. An increase in the particle sizes was noted, from 307 nm to 728 nm (Table 2), which was in accordance with the proposed hypothesis.

## 3. Materials and Methods

### 3.1. Materials

Chitosan (CHT) from crab shells (practical grade; Sigma–Aldrich, Darmstadt, Germany) with a molecular weight in the range of 190,000–310,000 Da, and ca. 85% deacetylation was used after purification by a reprecipitation method [45]. Virgin coconut oil (VCO), a commercial product, was purchased from Copra Indústria Alimentícia Ltd.a, Maceió, Brazil. The composition of the VCO’s main saturated fatty acids and their percentage presence (%) was provided by the manufacturer, as follows: lauric acid, C12-43.5%; myristic acid, C14-18.4%; palmitic acid, C16-10.3%; oleic acid, C18-8.6%; caprylic acid, C8-6.8%; stearic acid, C18-2.7%; capric acid, C10-5.4%; and caproic acid, C2-0.5%. In addition, Nile red dye (9-(diethylamino)benzo[a]phenoxazin-5(5H)-one) was purchased from Sigma-Aldrich Pty. Ltd. (Castle Hill, Australia). All other chemicals were reagent grade and were used as received. Cosmetic grade phytantriol (3,7,11,15-tetramethyl-1,2,3-hexadecanetriol) with 97% purity was produced by the DSM company and purchased from Alianza Magistral (São Paulo, Brazil). Pluronic^®^F127 (F127), Nile Red (NR), phosphate-buffered saline (PBS) at 1 mM, sodium diclofenac (SD), and Amicon Ultra-4 filters (10,000 MW, Millipore) were purchased from Merck-Sigma-Aldrich, Brazil. Deionized water (Milli-Q, Millipore Corp., Bedford, MA, USA) was used to prepare all aqueous samples.

### 3.2. Methods

#### 3.2.1. Cubosome Preparation

Cubosomes were produced following the protocol described in the literature [46]. Briefly, 0.2 g of phytantriol was weighed into a vial and heated at 40 °C until free flowing. Next, 1.2 mL of F127 stock solution of 5.0% (*w*/*v*) in phosphate buffer solution (PBS) was added and vortexed (1 min), and 8.8 mL of PBS was added to the vial. Phytantriol and F127 were used at a final concentration of 2.0% (*w*/*v*) and 0.6% (*w*/*v*), respectively. The mixture was dispersed using tip-ultrasonication (tip of 3 mm) at 500 W with an Ultrasonic Processor VCX series (Sonic & Materials Inc., Newtown, CT, USA). The amplitude was set at 40% from 500 W, and the pulse cycle consisted of 15 s ON and 3 s OFF for 30 min. A milky cubosome dispersion resulted from this process. A similar procedure was used to produce cubosomes containing SD, including 0.2% (*w*/*v*) SD.

#### 3.2.2. Preparation of CHT/VCO-Based Emulsion Scaffolds

**Scheme 1:**
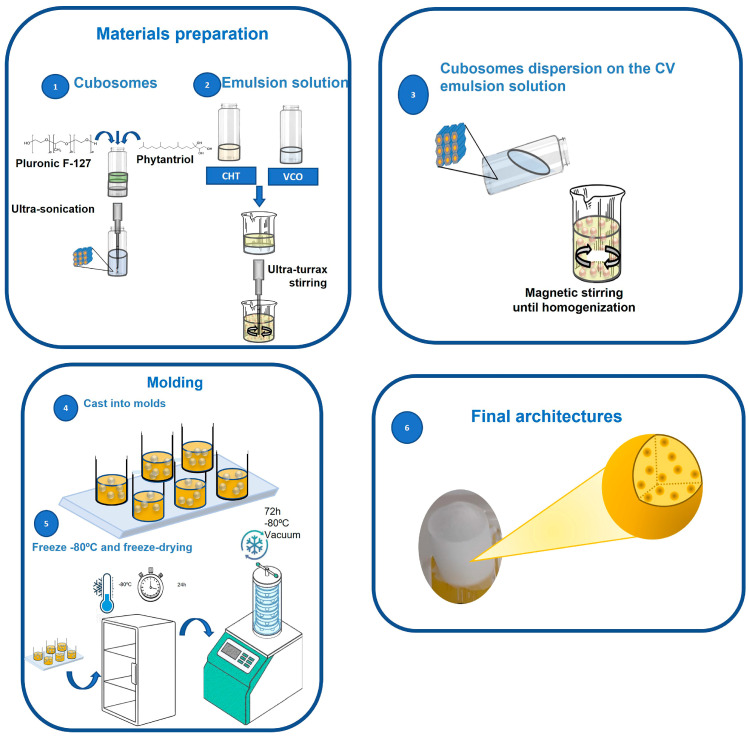
Schematic representation of the preparation of CV-based scaffolds doped with cubosomes.

A CHT solution at a concentration of 3% (*w*/*v*) was prepared in acetic acid solution (1% *v*/*v*), under constant stirring at room temperature overnight. Further, the solution was filtered through a sintered glass filter to eliminate impurities. To create the emulsion systems, VCO 1% (*v*/*v*) and glycerol 1% (*v*/*v*) were separately added to the CHT solution, and the systems were homogenized at 10,000 rpm for 10 min using a high-speed homogenizer (Ultra Turrax, T18 Basic, IKA-Werke GmbH & Co. KG, Staufen im Breisgau, Germany)., as shown in Scheme 1. Further, empty cubosomes and cubosomes loaded with diclofenac (0.1 mL) were added to the CHT/VCO (9.9 mL) under gentle stirring at room temperature to immobilize the cubosomes into the systems. The CHT/VCO was designed as CV throughout the manuscript. Please see the experimental compositions in Table 3.

### 3.3. Characterization

#### 3.3.1. Dynamic Light-Scattering (DLS) Measurements

Mean diameters (Z-average) and polydispersity indices (PDI) values of the cubosome dispersions were determined using a dynamic light-scattering instrument (Zetasizer NanoZS, Malvern, Queijas, Portugal) with a laser of 632.8 nm at an angle of 173° and at 25 °C. The dispersions were previously diluted in PBS (5.5 times) to avoid multiple light scattering for size determination. The average results corresponded to three successive analyses (100 s each) for at least three different batches. The autocorrelation functions were analyzed by the cumulant method and the inverse Laplace transform-based CONTIN algorithm. The Z-average size and the PDI were considered only for samples displaying a monomodal size distribution.

#### 3.3.2. Small-Angle X-ray-Scattering (SAXS) Measurements

The measurements were performed at the Laboratory of Complex Fluids, Institute of Physics (IF), University of São Paulo (USP), with SAXS equipment (Xenocs XEUSS™, MA, USA), using X-ray radiation generated by a source GENIX™, Xenocs, Holyoke, MA, USA (Cu Kα, λ = 1.54 Å) with a spot size of 0.7 mm × 0.7 mm, which was large enough to sample many nanoparticles at random orientations. The detector was a Dectris Pilatus™ (Philadelphia, PA, USA) 300 k with a 1.2 m sample-to-detector distance. The scattering angle (2θ) ranged between 0.12° and 4.59°, and the resulting q values ranged from 0.09 to 3.27 nm^−1^. The measurements were performed for empty cubosomes and cubosomes containing sodium diclofenac, both in PBS and for the CV-developed scaffolds.

Based on the relative peak positions in the SAXS curves (q), the cell parameters (a) were determined for the liquid crystalline dispersions in relation to the Miller indices (hkl), using Equation (1):

(1) $${\left(\frac{q}{2\pi }\right)}^{2}={\left(\frac{1}{a}\right)}^{2}\left(h^{2}+k^{2}+l^{2}\right)$$

The results were acquired in four frames of 30 s for each sample. These dispersions were measured in air and inside thin-walled boron-rich capillary glass tubes with 0.1 mm of thickness. Before the analysis, the samples (CV, CV_Cub, and CV_CubD) were swollen in PBS for 24 h. Four frames of 15 s were acquired for each sample.

#### 3.3.3. Scanning Electron Microscope

The morphology of the samples was examined using a NanoSEM-FEI Nova 200 (FEG/SEM, JSM-6010 LV, JEOL, Tokyo, Japan) scanning electron microscope. Before SEM analysis, the scaffolds were coated with gold using a Quorum/Polaron model E 6700 (East Sussex, UK) equipment. The analysis was performed with an acceleration voltage of 5.00 kV, and magnification ranged from 150× to 4000×. Moreover, the pore size of the scaffolds was determined using the software ImageJ, version 1.53t (Bethesda, MD, USA).

#### 3.3.4. Fourier Transform Infrared Spectroscopy

The chemical characterization of the developed scaffolds was performed using a Fourier transforms infrared (FTIR) obtained using a Shimadzu-IR, Prestige 21 spectrometer (Kyoto, Japan) equipped with a single horizontal ATR cell. The spectrum was collected by averaging 32 scans with a resolution of 4 cm^−1^, in the spectral region of 4000–400 cm^−1^.

#### 3.3.5. Mechanical Properties

The mechanical behavior of the sponges was performed under compression load using a Universal Mechanical Testing Equipment Instron 5543 (Norwood, MA, USA), equipped with a 50 N load cell at room temperature. The crosshead speed was set as 2 mm·min^−1^, and at least 6 specimens with a cylindrical shape of 6 mm in diameter and 10 mm of height were used for each sponge composition. The determination of the elastic modulus was used as the initial slope in the stress-strain curve. The yield strength, or the yield point, was also accessed to describe the stress at the transition from elastic to plastic deformation, and it was determined by using the intersection of a parallel line to the modulus at offset strain of 0.2%.

#### 3.3.6. Swelling

The swelling tests were performed by immersing the CV, CV_Cub, and CV_CubD scaffolds in phosphate buffer solution (PBS, pH 7.4) and pH 5, for up to 5 days, at 37 °C, in triplicate. The weight of the swollen samples was determined after removing the excess surface water by gently tapping the surface with filter paper. The percentage of water uptake was determined using Equation (2), where Wi is the dry weight and Wf is the swollen sample.

(2) $$Water\;uptake=\frac{Wf-Wi}{Wi}\times 100$$

#### 3.3.7. Diclofenac-Release Quantification

Like the water-uptake procedure earlier described, the structures were soaked at 37 °C in PBS, which was used as incubation media (pH 7), for 2 days, under mild agitation to improve the diffusion through the incubating media. At predefined time points, the surrounding buffer media were removed from each container, and the same volume of PBS was added. The experiments were performed four times in triplicate (n = 12). The surrounding buffer media volume was maintained constant throughout the experiment.

A calibration curve was constructed based on six diclofenac concentrations, between 0.1 µg.mL^−1^ and 10.0 µg.mL^−1^, in PBS, from which absorbance was measured at 283 nm [47]. The absorbance of the retrieved samples was measured, and the achieved values were comparatively studied within a previously constructed calibration curve.

#### 3.3.8. Confocal Analysis

The oil droplets int the CV-based emulsion (CV and CV_Cub) were evaluated using Nile red staining, adapted from different protocols [44,48]. For this purpose, a stock solution of Nile red (NR), 9-diethylamino-5H-benzo[a]phenoxazine-5-one (0.1 mg/mL), was prepared in PBS and stored while protected from light. Nile red is an excellent vital stain for detecting intracellular lipid droplets by fluorescence microscopy and flow cytofluorometry [44]. NR dilute solution (1:1000, 10 µL) was added to cubosomes in PBS and CV-based emulsion systems. The systems were stirred for 2 min and incubated for 20 min at 4 °C. Fluorescence images from stained systems were obtained using a confocal laser scanning microscope (Airyscan LSM980, Zeiss, Jena, Germany). The oil-soluble Nile red can be excited at 552 nm and then emits above 600 nm. Finally, the size of the droplets were determined using the software ImageJ.

### 3.4. Statistical Analysis

All the performed tests were performed in triplicate, and the obtained data points are presented as mean values and as the respective standard deviations. Statistical analysis was performed with GraphPad Prism 8.0 software (San Diego, CA, USA). The data were analyzed using Tukey’s multiple comparisons test to determine statistical differences. The significance level between the groups was set for * *p* < 0.05, ** *p* < 0.01, and *** *p* < 0.005. The data were shown as mean ± standard deviation (SD).

## 4. Conclusions

This study successfully demonstrated the production of 3D porous structures by combining chitosan and virgin coconut oil. Furthermore, the system supported the incorporation of doped cubosomes, without compromising the system’s physico-chemical features, allowing the development of a versatile drug-delivery platform to benefit from its components’ synergistic action. Phytantriol cubosomes were successfully dispersed in the emulsion-based solutions, presenting a homogeneous distribution with no visible nanoparticle aggregation. 

These findings indicated that the chemical environment of the emulsion was not significantly influenced by the cubosome dispersion in the solution, maintaining the chemical stability of the reported emulsion system. In addition, it was possible to observe that, in the presence of the structures loaded with diclofenac, the mechanical stability of the scaffolds was slightly improved or not affected, indicating appropriate support for drug delivery. Moreover, the release of the model drug, diclofenac, was favored by the migration of short-chain VCO fatty acids from the micelles to the cubosomes, which reduced the influence of the electrostatic interactions between the drug and the cubosome. 

Synergistically, the presence of the oil in the 3D architecture favored the diffusion of the drug through the structure, due to its lipophilic nature, which significantly improved the efficiency of the delivery of the used model drug. These findings demonstrated the versatility of CV-based systems for biomedical applications and explored the potential of cubosome nanoparticles to encapsulate active agents for drug release and targeted drug-delivery systems, especially for drugs with a lipophilic nature.

## Figures and Tables

**Table 1:** Mean diameter of cubosome dispersions in PBS for cubosomes (empty) and cubosomes loaded with 10 wt.% of diclofenac with respect to phytantriol, and for cubosomes after 48 h of releasing from scaffolds.

Cub	Mean Diameter (nm)
Cub	215 ± 30
Cub_D	215 ± 12
CV_Cub ^1^	550 ± 37
CV_CubD ^1^	748 ± 90

^1^ 48 h of release.

**Table 2:** Mean diameter of cubosome and/or oil droplets on cubosomes dispersion (Cub_NR) and emulsion solutions for CV_NR and CV_Cub_NR, obtained from confocal imaging.

Sample	Mean Diameter	
	Cubosomes (nm)	Oil Droplets (µm)
Cub_NR	307.8 ± 28.2	-
CV_NR	-	3.43 ± 1.01
CV_Cub_NR	728.3 ± 112.0	3.00 ± 0.97

**Table 3:** Experimental compositions of the CV-based scaffolds.

Identification	CHT (%)	VCO (%)	Gly (%)	Cubosomes (%)	Diclofenac (%)	Loaded Cubosomes (%)
CV	98	1	1	_	_	_
CV_Cub	97	1	1	1	_	_
CV_CubD	97	1	1	_	_	1
CV_D	97	1	1	_	1	_

## Data Availability

Research data are not shared.

## Supplementary Materials

Supporting information can be downloaded at: https://www.mdpi.com/article/10.3390/md21070394/s1, Figure S1: Degree of the swelling of the CV-based scaffolds immersed in pH 5. The dashed lines meaning the two-maximum degree of swelling for the different formulations for comparison proposes.
