# Supplementary material for: Chitosan/Virgin-Coconut-Oil-Based System Enriched with Cubosomes: A 3D Drug-Delivery Approach
Source: Mar Drugs. 2023 Jul 6;21(7):394. doi: 10.3390/md21070394 (PMC10381190; doi:10.3390/md21070394)
Supplement: Supplementary file 1 [file marinedrugs-21-00394-s001.zip › marinedrugs-2477198-supplementary.pdf]

Supplementary information

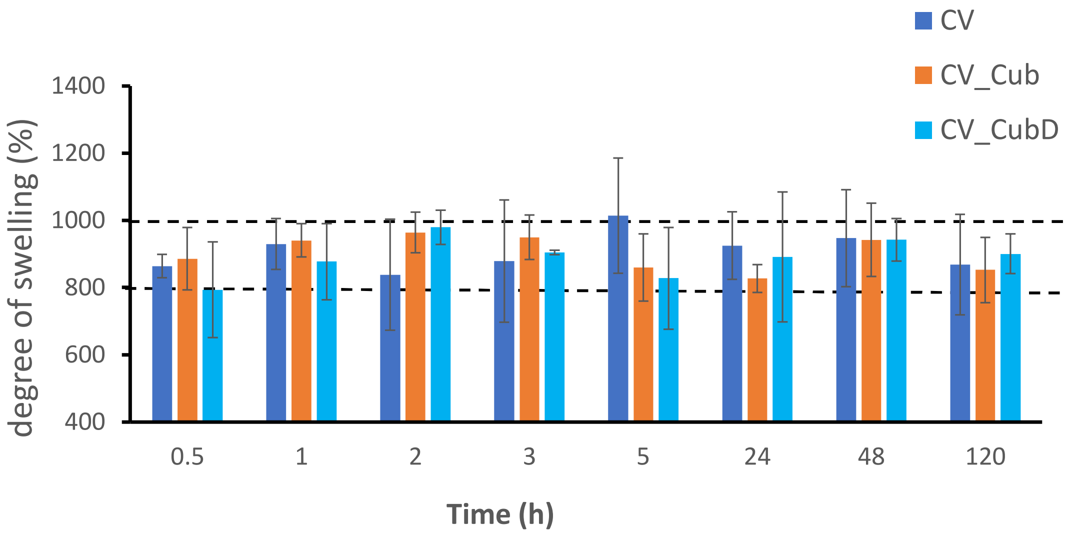

Figure S1. Degree of the swelling of the CV-based scaffolds immersed in pH5. The dashed lines meaning the two-maximum degree of swelling for the different formulations for comparison proposes.
